# Understanding of cervical cancer, human papillomavirus (HPV) and HPV vaccine among women from Pakistan and Afghanistan

**DOI:** 10.3332/ecancer.2025.1891

**Published:** 2025-04-15

**Authors:** Gao Luwen, Hira Hameed, Bilal Aslam, Zhou Liyan, Abdul Jabbar, Anum Syyam

**Affiliations:** 1 Jiangxi Medical College, Shangrao, Jiangxi Province, China; 2 The Islamia University of Bahawalpur, Bahawalpur 63100, Punjab, Pakistan; 3 Department of Veterinary Preventive Medicine, College of Veterinary Medicine, Qassim University, Buraydah 51452, Saudi Arabia; 4 Institute of Microbiology, Government College University Faisalabad, Faisalabad, Pakistan; 5 Department of Veterinary Science, University of Veterinary and Animal Sciences Lahore, Lahore 5400, Pakistan; 6 Department of Allied Health Sciences, International Institute of Science, Arts and Technology (IISAT), Gujranwala 52250, Pakistan

**Keywords:** cervical cancer, HPV, vaccination, knowledge, Afghan, Pakistan

## Abstract

Cervical cancer, primarily caused by persistent infection with high-risk strains of Human Papillomavirus (HPV), is a major health concern worldwide, particularly in developing countries like Pakistan and Afghanistan. A total of 212 women participated in the study, with 68.4% from Pakistan and 31.6% from Afghanistan. The majority of participants (80.7%) were from urban areas, and the most common age group was 31 to 50 years (46.2%), 60.8% being illiterate, 77.3% were non-working and 92% were married. The study found that both Pakistani and Afghan participants had similar, non-significant knowledge regarding cervical cancer and HPV, with Pakistani participants showing slightly higher awareness. Education played a crucial role in knowledge levels; Pakistani women, particularly those with higher education, were more informed about the prevention and early treatment of cervical cancer. However, knowledge about HPV vaccines was low across both groups, with Pakistani women being more aware than Afghan women. In terms of willingness to vaccinate children and pay for vaccines, Pakistani participants showed greater enthusiasm compared to Afghan participants, although the differences were non-significant. Logistic regression analysis showed that education, urban background and employment status were significantly linked to knowledge about cervical cancer, HPV and its vaccine. Women with higher education and urban backgrounds had better knowledge and were more willing to pay for the vaccine. In conclusion, this study highlights the influence of socio-demographic factors, such as education, urban background and employment status, on women’s knowledge about cervical cancer, HPV and its vaccine. Although both Pakistani and Afghan participants demonstrated similar levels of awareness, Pakistani women, especially those with higher education, were more knowledgeable about prevention and early treatment options. Despite limited awareness about HPV vaccines, Pakistani participants exhibited more willingness to vaccinate their children and pay for the vaccine compared to Afghan participants. These findings underscore the importance of education and urbanisation in improving health knowledge and vaccine uptake.

## Introduction

Cervical cancer poses a significant public health challenge globally, particularly in regions with limited access to preventive healthcare services [1]. In Pakistan and Afghanistan, cervical cancer is a leading cause of morbidity and mortality among women [2]. Human Papillomavirus (HPV) infection is the primary etiological factor for cervical cancer and vaccination against HPV is effective in preventing infection and subsequent development of cervical cancer. However, knowledge and awareness about cervical cancer, HPV and the HPV vaccine among women in these regions remain limited [3]. Understanding the level of knowledge and awareness among Pakistani and Afghan women regarding cervical cancer, HPV and the HPV vaccine is crucial for designing effective prevention and intervention strategies to combat the burden of cervical cancer in these populations [4, 5].

With an estimated 604,000 new cases and 342,000 deaths globally in 2020, cervical cancer is the third most common malignancy among women [6]. Due to the widespread use of cervical screening tests and immunisations, developed nations have seen a decrease in the incidence and fatality rates of cervical cancer [7]. HPV infection is one of the most prevalent sexually transmitted infections of the genital system and is known to be a significant cause (99.9%) of cervical cancer [8]. Developing nations account for 88% of cervical cancer-related fatalities and 85% of infections [9]. The World Health Organisation (WHO) (2022) estimated that several factors, including limited access to healthcare facilities, policymakers, healthcare workers and the general public’s lack of knowledge about cervical cancer, contribute to higher rates of cervical cancer prevalence and mortality in developing countries [10]. It is also believed that in many nations, women have relatively little awareness regarding HPV and cervical cancer [11].

With a few notable exceptions, most Pakistani and Afghani women are quite ready to vaccinate their children against HPV and cervical cancer, despite differences in their understanding and views about these issues [12]. There is a severe lack of knowledge and awareness of HPV, cervical cancer and the effectiveness of the HPV vaccination in preventing cervical cancer worldwide, particularly in Pakistan [13]. Research has shown that there are significant differences in the level of knowledge of HPV and cervical cancer among various communities in Pakistan [14]. The HPV vaccination, which should be given before a person has their first sexual experience, has been approved by the WHO as the main strategy for preventing cervical cancer [15]. Since the current vaccinations are shown to be successful in preventing genital warts and anal pre-cancers in both sexes, several countries have now started immunising guys against HPV [16]. With almost 14 million cases worldwide in 2008, the prevalence of HPV-associated infections was very high [17]. A 2013 WHO study states that cervical cancer is the greatest cause of mortality worldwide, accounting for around 0.27 million deaths annually and almost 660,000 new cases in 2022, cervical cancer ranks fourth among cancers that affect women worldwide. Around 94% of the 350,000 cervical cancer-related fatalities that year happened in low- and middle-income nations [18]. Because of poor and insufficient access to screening and treatment, 85% of these fatalities occur in middle-class or lower-income nations [19].

The purpose of the present research was to evaluate the awareness of cervical cancer, HPV and HPV vaccination. There is very little awareness and research conducted in Pakistan and Afghanistan about these disastrous diseases which can be fatal among women along newborns. Knowledge of cervical cancer, HPV and the HPV vaccination among Pakistani and Afghani women is divided into two categories: (1) knowledge of HPV-positive vs HPV negative women; and (2) variables related to knowledge of HPV, the HPV vaccine and HPV-associated malignancies, with the ultimate goal of informing targeted education and vaccination campaigns to reduce the incidence of cervical cancer in these regions.

## Methods

### Ethical statement

The study protocol adhered to the principles outlined in the Declaration of Helsinki and received approval from the Ethics Committee of Government College University Faisalabad, Pakistan. Informed written consent was obtained from each participant individually.

### Participant recruitment

This cross-sectional study was conducted to assess the knowledge and awareness of cervical cancer, HPV and the HPV vaccine among women from Pakistan and Afghanistan. The study included women aged 18 to 65 years residing in various regions of Pakistan and Afghanistan. Convenience sampling was utilised to recruit participants from diverse socioeconomic backgrounds and geographic locations within the two countries. Participants were provided with information about the study objectives and procedures, and informed consent was obtained before data collection. For this study, the sample size was calculated based on previous studies [20–22].

### Questionnaire data and process

A structured questionnaire was developed based on a review of the literature and a previously published questionnaire and expert input [20–22]. The questionnaire included sections on demographic characteristics, knowledge about cervical cancer and HPV, sources of information and attitudes toward HPV vaccination. Trained interviewers administered the questionnaire face-to-face or via telephone interviews, depending on participant preference and accessibility.

### Statistical analysis

The data collected from participants were inputted into a database, and both descriptive statistics were computed using SPSS version 20.0 for Windows (SPSS Inc., Chicago, IL). Descriptive statistics, including frequencies and percentages, were used to summarise participants’ responses. Participants were stratified into subgroups based on their regional backgrounds to examine the associations between cervical cancer, and HPV-related knowledge, attitudes, behaviors and intentions. Chi-square tests were employed to assess differences across various groups. Awareness and knowledge regarding cervical cancer, HPV and the HPV vaccine were presented as percentages. Logistic regression analysis was conducted to assess the role of different variables in influencing knowledge and awareness about cervical cancer and HPV. Cervical cancer and HPV knowledge served as independent variables, while other factors such as ethnicity, region, education, occupation, monthly income and age were treated as dependent variables. All independent variables were categorised into two or more groups. All statistical tests were two-sided, and *p* values <0.05 were considered statistically significant.

## Results

A total of 212 women, (68.4%) from Pakistan and (31.6%) from Afghanistan participated in the study. There were (80.7%) of women belonged to urban and (19.3%) from rural areas. The participants’ ages varied from under 30 to over 50 years old. Age was classified into three age categories. The most common age group of study was 31 to 50 years old (46.2.5%). The remaining age categories consisted of 88 women over 50 years old (41.5%) and 26 women less than 30 years old (12.3%). The majority of participants were illiterate (60.8%), while (39.2%) had elementary or higher education. There were 165 non-workers (77.3%) and 47 workers (21.7%) among the participants. 45.3% of the participants had less than 3,000 Rupees income per month, while 37.7% had more than 3,000 Rupees income per month. Additionally, (17%) had no income. According to their marital status, 92% were married and 8%, were unmarried, respectively. According to the family system, there were 56.1% of women lived in separate homes, while 34.9% in a joint family system. The participants that had 1–5 family members were 24.1%, 6–10 members had (59.9%) and those that had >10 family members were 35 (16%). The demographic characteristics of the participants are summarised in Table 1.

From all the Pakistani and Afghani participants, both were known non-significantly (*p* > 0.05) about cervical cancer and HPV infection. They knew about its transmission and causes. Meanwhile, Pakistani participants (*n* = 144) were more aware of cervical cancer and HPV. All the participants of this study (*n* = 34) non-significantly knew about the fact that cervical cancer can be prevented and cured by early treatments. In this research, Pakistani respondents (*n* = 28) had higher levels of education than Afghani respondents (*n* = 6); hence, they were better knowledgeable about the early cure and prevention of cervical cancer (Table 2).

All the participants of this study (*n* = 49) non-significantly knew about HPV. From all the respondents, Pakistani participants (*n* = 37) knew more about HPV than Afghani (*n* = 12). All the participants (*n* = 16) non-significantly knew that HPV can be transmitted via sex. Uneducated people do not know about protected sex. Meanwhile, Pakistani-educated women (*n* = 13) have more knowledge about protected sex and the transmission of HPV via sex. While all participants (*n* = 20) did not significantly agree that HPV may cause genital warts, there was more discussion about Pakistani women (*n* = 16) than Afghani women (*n* = 4). All the participants (*n* = 14) significantly knew that HPV infection can lead to cervical cancer but only one Afghani woman knew about this (Table 2). Knowledge about HPV vaccines was very low among all participants (*n* = 33). Among them, Pakistani women (*n* = 26) had better knowledge about the HPV vaccine than Afghan women (*n* = 7). All participants (*n* = 80) showed a non-significant willingness to vaccinate their children, with Pakistanis (*n* = 58) being more eager than Afghanis (*n* = 22). Pakistanis (*n* = 21) tended to be more ready to pay for vaccinations than Afghans (*n* = 10), but all participants (*n* = 31) were not statistically different from one another in this regard (Table 2).

Logistic regression analysis was performed to assess the association between demographic factors and knowledge about cervical cancer (Table 3), knowledge about HPV (Table 4), HPV vaccine (Table 5) and the willingness to pay vaccine price (Table 6). Our findings revealed that knowledge about cervical cancer, HPV, its vaccine and willingness to pay for the vaccine was closely associated with socio-demographic characteristics in both groups of women. Women with higher levels of education and urban background demonstrated significantly greater knowledge and awareness regarding cervical cancer, and HPV compared to those with lower educational attainment and rural background. Additionally, urban, educated and non-working women were significantly more willing to pay for the vaccine than their counterparts.

## Discussion

Widespread HPV vaccination and inoculation are recognised to potentially reduce the incidence of cervical cancer. To the best of our knowledge, this research is the first of its kind to objectively measure women’s knowledge about HPV and cervical cancer in Pakistani and Afghani women, as well as to identify the cause and suggest vaccination. Many more people now live in cities than in tribal areas that were devastated by war as a result of internal displacement. All of these elements have led to a rise in the burden of disease on the population, an inadequate healthcare system, a lack of access to appropriate treatment and a lack of knowledge about different diseases. The majority of community health awareness campaigns in Pakistan have addressed issues such as hepatitis C virus, diabetes, obesity, tuberculosis and breast cancer. On the other hand, barely much research has been done on cervical cancer in Pakistani women [23]. Due to a lack of knowledge and restricted access to reliable screening, cervical cancer is not detected until it is symptomatic. There may be limited treatment options for such advanced diseases, which would lead to a higher cervical cancer mortality rate in these nations. According to our survey, the majority of participants knew very little about HPV and cervical cancer, particularly how to prevent and cure it. The result of this survey is similar to the study [24]. They also mostly failed to identify cervical cancer as a form of gynecological cancer.

In all, 212 women from Pakistan and Afghanistan were involved in the study. The findings indicate a very low level of overall knowledge and awareness about cervical cancer (22.6%); HPV (23.1) and HPV vaccines (15.5%) among all women. Furthermore, differences in knowledge and awareness about cervical cancer, HPV and its vaccines have been reported among Pakistan and Afghan women. This level of knowledge of very low compared to other countries women such as China [20] and Scandinavian women [25]. However, it is comparable with Senegalese adolescents, where only almost 26% of women know about HPV [26]. The age range of the participants was 31 to 50 years old. The majority of the participants lacked formal education, were not employed, earned less than 3,000 Rupees per month, majority participants were married and had six to ten family members, Most of the Pakistani women still have less knowledge about cervical cancer, HPV infection and its vaccination as described in Riaz *et al* [27]. Because in many areas where people have no education and have adopted an old lifestyle, they do not know about hygiene and protection from infections. Inefficient cervical cancer screening is a result of many problems in developing and low-income nations. One of them might be the lack of a nationwide program for cervical cancer screening among women, poorly designed healthcare services, relatively limited female access to healthcare facilities and a lack of technical expertise. All of these things lead to ineffective testing, delayed diagnosis and subpar care, which in turn raises the death rate for females. Enhancement in all the aspects mentioned above can only be successful if women and the general public are informed about cervical cancer, its causes and the available vaccinations. The high percentage of illiteracy and various religious beliefs further impede access to correct information in less developed nations. We recommend that the start of a high-level public education campaign on the HPV vaccine be given top priority to specifically address awareness gaps in the general public. Most women were not aware that early detection and screening may lessen the effects of cervical cancer as also explained in the study [28].

Pakistan, as a developing nation, faces significant challenges in addressing HPV-related health issues due to the absence of a routine HPV screening and vaccination program. Despite the growing global emphasis on HPV prevention and early detection, the country has yet to implement a standardised framework to combat this pressing health concern. Access to HPV testing remains extremely limited, with only a handful of hospitals across the nation equipped with the necessary facilities and diagnostic systems. This lack of infrastructure not only hinders early detection and timely intervention but also contributes to the rising burden of HPV-associated diseases, including cervical cancer. Establishing a comprehensive HPV screening and vaccination program in Pakistan is crucial to improving public health outcomes and reducing the long-term impact of HPV infections. Our research shows that the participant’s knowledge of HPV vaccinations was inadequate. The percentage of women who agreed to take a vaccination was quite low (15.05%). Almost all of them also expressed a desire to learn more about the vaccine and were open to participating in cancer prevention and awareness campaigns. According to our findings, participants like those in other developing nations [29] have good opinions regarding receiving the HPV vaccination. Our findings suggest that even the educated people in Pakistan do not know the basic facts regarding cervical cancer caused by HPV and the vaccines that prevent it. A small number of epidemiological studies carried out in the past in various regions of Pakistan have shown that HPV is a significant cause of cervical cancer in Pakistani women [30, 31].

Several limitations in this study may have an impact on the findings. First, cross-sectional data restrained the ability to determine whether variations in awareness levels depended on demographic/socioeconomic factors. Second, the number of participants included 212 women only and enough number may not be a probability sample to generalise about all the women in Pakistan and Afghanistan especially due to significant differences between Urban/Rural women and those who are Educated/Illiterate women. Third, the use of self-administered questionnaires may have response bias where respondents may provide an inflated awareness score due to respondent bias resulting from the socially desirable response bias of respondents or inability to understand survey questions. In the same regard, the convenience sampling approach could have led to the omission of some subgroups, for instance, persons from extremely remote areas, who could have comparatively lesser awareness regarding, and access to health education. Last but not least; practical barriers and culture prevented the extent of probing the participants on some crucial issues including sexual health and transmission of HPV.

Further research should be oriented to eliminating these shortcomings by employing larger samples, selecting, as much as possible, to represent the population and the use of longitudinal research designs focusing on changes in awareness and attitudes in the future. It is also important to note that increasing the types of participants of the study to men and healthcare providers would provide a broader perspective and comprehensive understanding of community-level barriers to HPV prevention and cervical cancer screening. Furthermore, there is a definite need to introduce specific alterations regarding community integrative educational programs for awareness, and culturally sensitive awareness-raising crusades. Such activities should foster an understanding of HPV vaccination and cervical cancer screening possibly leading to a decrease in disease incidence in these areas. Policymakers may apply these findings to create progressively more health-inclusionary approaches, increase accessibility to vaccinations, as well as call for the creation of national cervical screening programs.

## Conclusion

In conclusion, we discovered a considerable amount of regional variance in the knowledge and awareness of cervical cancer, HPV and HPV vaccines. Compared to women from Pakistan and Afghanistan, Pakistani women were more informed about cervical cancer and HPV. There was relatively little knowledge of HPV, cervical cancer and the HPV vaccine, despite a moderate degree of awareness and comprehension of the disease. It is critical to focus outreach on populations, where there are information gaps to encourage discussion about vaccination between patients and their medical professionals. Our research also demonstrates the need to create and execute public education campaigns to inform women about cervical cancer, HPV, HPV vaccination and its implications.

## Conflicts of interest

The authors declare no conflicts of interest.

## Funding

No funding

## Author contributions

GL and AJ conceived the idea of study. HH, BA and ZL performed experimental work. AJ wrote the manuscript. GL and AS helped in the data collection and writing of the manuscript. GL, HH, BA, ZL, AJ and AS proofread the article.

## Figures and Tables

**Table 1.** Demographic characteristics of Pakistan and Afghan women participants.

Characteristics	Frequency	Percent
Nationality		
Pakistani	144	68.4
Afghan	68	31.6
Area		
Urban	172	80.7
Rural	40	19.3
Religion		
Islam	203	95.3
Other	9	4.7
Age		
<30	26	12.3
31–50	98	46.2
>50	88	41.5
Education		
Illiterate	128	60.8
Primary or more	84	39.2
Occupation		
Non worker	165	77.3
Working	47	21.7
Monthly income		
<3,000/month	96	45.3
>3,000/month	80	37.7
No income	36	17.0
Marital status		
Single	17	8.0
Married	195	92.0
Family system		
Single	118	56.1
Joint	94	43.9
Family member		
1–5 person	51	24.1
6–10 person	126	59.9
>10 person	35	16.0

**Table 2.** Levels of knowledge about cervical cancer and HPV among Pakistan and Afghan women.

Variables	Total	Pakistan (*n* = 144)	Afghan (*n* = 68)	*p*-value
Do you know about cervical cancer?	48 (22.6)	36 (25.0)	12 (17.6)	0.29
Knows it can be cured by early treatment	34 (16.0)	28 (19.4)	6 (8.8)	**0.05**
Knows about HPV	49 (23.1)	37 (25.7)	12 (17.6)	0.19
Knows HPV can be transmitted via sex	16 (7.5)	13 (9.0)	3 (4.4)	0.23
Knows that HPV can cause genital warts	20 (9.4)	16 (11.1)	4 (5.9)	0.22
Knows that HPV infection can lead to cervical cancer	14 (6.6)	13 (9.0)	1 (1.5)	**0.03**
Knows about the HPV vaccine	33 (15.5)	26 (18.0)	7 (10.3)	0.2
Received HPV vaccination	0	0	0	-
Willing to vaccinate children	80 (37.8)	58 (40.7)	22 (32.3)	0.26
Willing to pay for vaccination	31 (14.6)	21 (14.6)	10 (14.7)	0.9

**Table 3.** Logistic regression analysis of the levels of knowledge about cervical cancer.

Variables	Total	Knowledge	OR (95%)	*p*-value
Nationality				0.6
Pakistani	144	36 (25.0)	1	
Afghan	68	12 (13.6)	0.8 (0.4–1.9)	
Area				0.3
Urban	172	40 (23.2)	1	
Rural	40	8 (20.0)	0.6 (0.2–1.7)	
Religion				0.9
Islam	203	46 (22.7)	1	
Other	9	2 (22.2)	1.0 (0.2–5.6)	
Age				0.5
<30	26	4 (15.4)	1	
31–50	98	25 (25.5)	0.7 (0.1–3.4)	
>50	88	19 (19.2)	0.5 (0.1–2.4)	
Education				**0.05**
Illiterate	128	23 (18.0)	1	
Primary or more	84	25 (29.8)	2.1 (1.0–4.4)	
Occupation				0.7
Non-worker	165	37 (22.4)	1	
Working	47	11 (23.4)	0.8(0.2–3.4)	
Monthly income				0.7
<3,000/month	96	23 (23.9)	1	
>3,000/month	80	16 (20.0)	0.7 (0.3–1.7)	
No income	36	9 (25.0)	0.9 (0.2–4.4)	
Marital Status				0.1
Single	17	1 (5.9)	1	
Married	195	47 (24.1)	7.5 (0.6–99.5)	
Family system				0.7
Single	118	24 (20.3)	1	
Joint	94	24 (25.5)	1.2 (0.5–3.0)	
Family member				0.6
1–5 person	51	13 (25.5)	1	
6–10 person	126	25 (19.8)	0.7 (0.3–1.9)	
>10 person	35	10 (28.6)	1.1 (0.3–4.5)	

**Table 4.** Logistic regression analysis of the levels of knowledge about HPV.

Variables	Total	Knowledge	OR (95%)	*p*-value
Nationality				0.7
Pakistani	144	37 (25.7)	1	
Afghan	68	12 (13.6)	0.8 (0.4–2.0)	
Area				**0.07**
Urban	172	43 (25.0)	1	
Rural	40	6 (15.0)	0.3 (0.1–1.1)	
Religion				0.9
Islam	203	47 (23.1)	1	
Other	9	2 (22.2)	1.1 (0.2–6.4)	
Age				0.5
<30	26	4 (15.4)	1	
31–50	98	26 (26.5)	0.7 (0.1–3.4)	
>50	88	19 (19.2)	0.4 (0.1–2.32)	
Education				**0.03**
Illiterate	128	24 (18.7)	1	
Primary or more	84	25 (29.8)	2.3 (1.0–4.9)	
Occupation				0.7
Non-worker	165	39 (23.6)	1	
Working	47	10 (21.3)	0.7 (0.2–3.3)	
Monthly income				0.6
<3,000/month	96	24 (25.0)	1	
>3,000/month	80	17 (21.2)	0.6 (0.2–1.5)	
No income	36	8 (22.2)	0.9 (0.2–4.3)	
Marital status				0.1
Single	17	1 (5.9)	1	
Married	195	48 (24.6)	8.8 (0.6–118.7)	
Family system				0.8
Single	118	24 (20.3)	1	
Joint	94	25 (24.6)	1.1 (0.4–2.7)	
Family member				0.7
1–5 person	51	12 (23.5)	1	
6–10 person	126	27 (21.4)	0.9 (0.3–2.3)	
>10 person	35	10 (28.6)	1.3 (0.3–5.2)	

**Table 5.** Logistic regression analysis of the levels of knowledge about HPV vaccine.

Variables	Total	Knowledge	OR (95%)	*p*-value
Nationality				0.6
Pakistani	144	26 (18.0)	1	
Afghan	68	7 (7.9)	0.7 (0.3–2.0)	
Area				0.2
Urban	172	28 (19.7)	1	
Rural	40	5 (12.5)	0.5 (0.1–1.6)	
Religion				0.8
Islam	203	32 (15.8)	1	
Other	9	1 (11.1)	0.8 (0.9–8.0)	
Age				0.2
<30	26	3 (11.5)	1	
31–50	98	19 (19.4)	1.2 (0.2–7.1)	
>50	88	11 (11.1)	0.5 (0.1–2.9)	
Education				0.1
Illiterate	128	17 (13.3)	1	
Primary or more	84	16 (19.0)	1.9 (0.8–4.7)	
Occupation				0.4
Non-worker	165	24 (14.5)	1	
Working	47	9 (19.1)	1.8 (0.3–9.9)	
Monthly income				0.5
<3,000/month	96	17 (17.7)	1	
>3,000/month	80	9 (11.2)	0.5 (0.1–1.5)	
No income	36	7 (19.4)	0.6 (0.1–3.4)	
Marital status				0.4
Single	17	1 (5.9)	1	
Married	195	32 (16.4)	3.4 (0.2–52.3)	
Family system				0.9
Single	118	17 (14.4)	1	
Joint	94	16 (17.0)	1.1 (0.3–3.4)	
Family member				0.21
1–5 person	51	11 (21.6)	1	
6–10 person	126	14 (11.1)	0.5 (0.1–1.6)	
>10 person	35	8 (22.8)	1.0 (0.2–5.2)	

**Table 6.** Logistic regression analysis of the willingness to pay vaccine price.

Variables	Total	Knowledge	OR (95%)	*p*-value
Nationality				0.3
Pakistani	144	21 (14.6)	1	
Afghan	68	10 (14.7)	1.6 (0.6–4.5)	
Area				**0.03**
Urban	172	28 (16.3)	1	
Rural	40	3 (7.5)	0.2 (0.04–0.9)	
Religion				0.5
Islam	203	29 (14.3)	1	
Other	9	2 (22.2)	1.9 (0.3–12.2)	
Age				**-**
<30	26	1 (3.8)	1	
31–50	98	15 (15.3)	-	
>50	88	15 (17.0)	-	
Education				**0.03**
Illiterate	128	14 (10.9)	1	
Primary or more	84	17 (20.2)	2.9 (0.009–1.4)	
Occupation				**0.08**
Non-worker	165	27 (16.4)	1	
Working	47	4 (8.5)	0.1 (0.009–1.4)	
Monthly income				0.4
<3,000/month	96	14 (14.6)	1	
>3,000/month	80	13 (16.2)	0.6 (0.2–1.7)	
No income	36	4 (11.1)	2.6 (0.2–29.9)	
Marital status				**-**
Single	17	1 (5.9)	1	
Married	195	30	-	
Family system				0.8
Single	118	17 (14.4)	1	
Joint	94	14 (15.4)	0.8 (0.3–2.5)	
Family member				0.9
1–5 person	51	7 (13.7)	1	
6–10 person	126	19 (15.1)	1.0 (0.3–3.1)	
>10 person	35	5 (14.2)	1.2 (0.2–6.4)	
